# Can primary palliative care education change life-sustaining treatment intensity of older adults at the end of life? A retrospective study

**DOI:** 10.1186/s12904-021-00783-6

**Published:** 2021-06-21

**Authors:** Qian Liu, Mingzhao Qin, Jian Zhou, Hui Zheng, Weiping Liu, Qi Shen

**Affiliations:** Department of Geriatrics, Beijing Tongren Hospital, Capital Medical University, Dongjiaominxiang No.1, Dongcheng District, Beijing, 100730 China

**Keywords:** Primary palliative care, End-of-life, Older adults, Life-sustaining treatment, Education

## Abstract

**Background:**

Palliative care education has been carried out in some hospitals and palliative care has gradually developed in mainland China. However, the clinical research is sparse and whether primary palliative care education influence treatment intensity of dying older adults is still unknown. This study aims to explore the changes to the intensity of end-of-life care in hospitalized older adults before and after the implementation of primary palliative care education.

**Methods:**

A retrospective study was conducted. Two hundred three decedents were included from Beijing Tongren Hospital’s department of geriatrics between January 1, 2014 to December 31, 2019. Patients were split into two cohorts with regards to the start of palliative care education. Patient demographics and clinical characteristics as well as analgesia use, medical resources use and provision of life-sustaining treatments were compared. We used a chi-square test to compare categorical variables, a *t* test to compare continuous variables with normal distributions and a Mann–Whitney U test for continuous variables with skewed distributions.

**Results:**

Of the total participants in the study, 157(77.3%) patients were male. The median age was 88 (interquartile range; Q1-Q3 83–93) and the majority of patients (*N* = 172, 84.7%) aged 80 years or older. The top 3 causes of death were malignant solid tumor (*N* = 74, 36.5%), infectious disease (*N* = 74, 36.5%), and cardiovascular disease (*N* = 23, 11.3%). Approximately two thirds died of non-cancer diseases. There was no significant difference in age, gender, cause of death and functional status between the two groups (*p* > 0.05). After primary palliative care education, pain controlling drugs were used more (*p* < 0.05), fewer patients received electric defibrillation, bag mask ventilation and vasopressors (*p* < 0.05). There was no change in the length of hospitalization, intensive care admissions, polypharmacy, use of broad-spectrum antibiotics, blood infusions, albumin infusions, nasogastric/nasoenteric tubes, parenteral nutrition, renal replacement and mechanical ventilation (*p* > 0.05).

**Conclusions:**

Primary palliative care education may promotes pain controlling drug use and DNR implementation. More efforts should be put on education about symptom assessment, prognostication, advance care planning, code status discussion in order to reduce acute medical care resource use and apply life-sustaining treatment appropriately.

## Background

The proportion and absolute number of older people in populations around the world are increasing dramatically. With continuous improvement of economic conditions and rapid development of medical technology, life expectancy is constantly rising, with most deaths occurring in people older than 70 in high-income countries [1]. However, death is still an inevitable consequence of being born. China has become an aging society since 1999. By the end of 2018, there were 167 million people aged 65 years or older, accounting for 11.9% of the total population [2]. This has resulted in increasing concerns around issues associated with aging. According to the 2015 Quality of Death Index published by the Economist Intelligence Unit, mainland China ranked 71 out of 80 countries with respect to the quality of palliative care [3]. There remains a huge gap between the supply and demand of hospice and palliative care. Health-care professionals need to have knowledge and skills to meet this growing demand [4]. Mainland China needs to develop palliative care training urgently to meet these increasing demands and improve the quality of end-of-life (EOL) care.

Palliative care benefits terminally ill patients and their families. However, an international shortage and geographic maldistribution of specialist palliative care (SPC) clinicians means that many patients lack access to it [5]. As part of palliative care delivery, we need to distinguish primary palliative care (PPC) from specialist palliative care. Representative skills of PPC include (1) basic management of pain and symptoms (2) basic management of depression and anxiety (3) basic discussion about prognosis, goals of treatment, suffering and code status [6]. All clinicians who care for seriously ill patients should be able to deliver PPC [7]. Therefore, geriatricians who are constantly facing older adults with severe and life-threatening illnesses, require proficient PPC skills. Currently, palliative care board certification is not available in mainland China and specialty palliative care cannot be delivered in most hospitals and institutions. Thus, carrying out PPC education in geriatric departments is important to promote the quality of EOL care for older adults.

It is often necessary to provide symptom control rather than curative treatment for older adults at the EOL. At the same time, futile life-sustaining treatments (LST) such as cardiopulmonary resuscitation (CPR) should be avoided. However, this is a complex problem which involves a multitude of factors including medical staff training, the condition of the patient, patient autonomy, patient family preferences, the national culture and so on. Reducing high intensity treatments that prolong pain improves the quality of death. Although there is no uniform definition of EOL care intensity [8, 9], a systematic review [10] presented three main domains: hospitalization (acute hospital, intensive care unit, emergency department), life-sustaining invasive procedures (resuscitation, intubation, mechanical ventilation, artificial feeding and dialysis) and potentially life-prolonging treatments (surgery, chemotherapy, radiation, medical imaging, transfusions). There are also studies which explore the appropriateness of CPR in the elderly at the EOL, effective methods of delivering do-not-resuscitate (DNR), and related factors of high intensity treatment [8, 11]. In addition to reducing these three domains of treatment intensity, we think that high quality EOL care should also encompass adequate symptom control. This includes sufficient pain control and treatments to relieve symptoms such as dyspnea. The patients should neither be under-treated nor over-treated.

Although palliative care has gradually developed in mainland China in recent years, the clinical research is sparse. We need to explore local models to improve the quality of death, this includes increased palliative care education [12]. PPC education has been carried out since January 2018 in our department. We have also been involved in palliative care teaching for medical students and specialized nurses. Palliative care education is effective in improving health professional student’s knowledge and attitudes toward palliative care [13]. To the best of our knowledge, there are no clinical studies on the effect of PPC education on EOL care intensity in a geriatrics department in mainland China. The aim of this study is to explore the changes to EOL care intensity of hospitalized older adults before and after the implementation of PPC education.

## Methods

### Study design, setting and participants

This is a retrospective analysis reporting the EOL care intensity change in the inpatient department of geriatrics of Beijing Tongren hospital before and after PPC education. We consecutively included deceased participants between January 1, 2014 and December 31, 2019.

### Data collection and measurements

Medical information was extracted from the hospital’s electronic medical records database. Medical records were reviewed separately by two of the authors. They extracted the following clinical information from each enrolled individual: (1) Age, gender, length of stay, cause of death, times of hospitalization in the past 12 months, Barthal ADL score and comorbidities. (2) ICU admission, pain controlling drug use, pleural puncture, peritoneal puncture or pericardial puncture, oral medication numbers in the last week of life. (3) Persons who sign the CPR and preferences of CPR. (4) Broad-spectrum antibiotic use, blood infusion, albumin use, operation, chemotherapy, radiotherapy, intubation, tracheostomy, feeding tube, kidney replacement therapy and mechanical ventilation. (5) Detailed CPR procedures including chest compressions, electrical defibrillation, bag mask ventilation, vasopressor and respiratory stimulant use.

We used the Charlson Comorbidity Index (CCI) to evaluate the chronic disease. Polypharmacy was defined as 5 or more oral agents. Causes of death were classified as solid cancer, infectious disease, cardiovascular disease, hematological disease, chronic obstructive pulmonary disease, cerebro-vascular disease, renal failure and other causes. The above causes were then divided into three categories:(1) cancer including malignant solid tumor and hematologic tumor (2) infectious disease (3) other disease including cardiovascular diseases, chronic obstructive pulmonary disease, end stage renal disease, cerebrovascular disease and other causes.

According to the start point of PPC education, we divided participants into two cohorts: (1) usual care group (January 1, 2014 to December 31, 2017) (2) PPC education group (January 1, 2018 to December 31, 2019). The LST intensity was compared between the two cohorts.

The specific content of PPC education includes: (1) Medical staff participating in the national education program every year (2) All staff training at hospital level (cancer pain management) and pain assessment required in medical records when using pain controlling drugs (3) Hosting education program and lectures (4) Participating in the postgraduate palliative care course as a teaching hospital and training hospice nurses of Chinese Nursing Society as clinical teaching base (5) Patient's advance care planning education.

### Statistical analysis

The continuous variables are presented as mean ± standard deviation or median and interquartile range(IQR). The category variables are presented as number and percentage. We used a chi-square test to compare categorical variables, a *t* test to compare continuous variables with normal distributions and a Mann–Whitney U test for continuous variables with skewed distributions. A two-tailed *p*-value < 0.05 was considered statistically significant. We used IBM SPSS Statistics (version 17.0, IBM Corp., USA) to conduct the analysis.

## Results

### Baseline characteristics

**Fig. 1:**
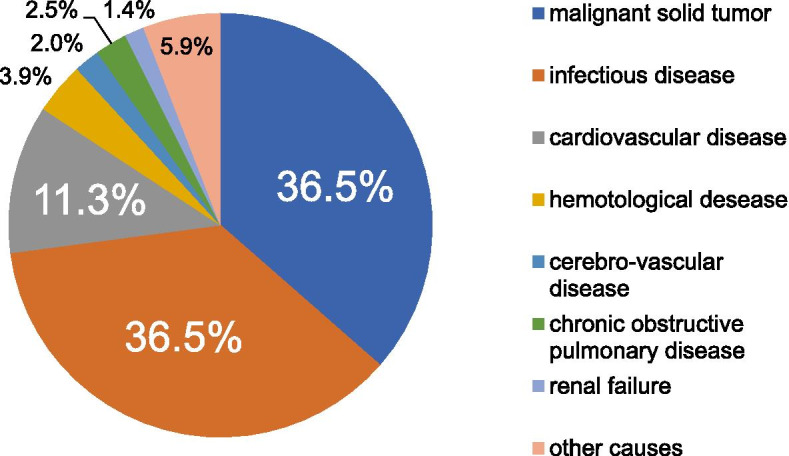
The death cause of all cases

Of the total 203 deceased patients in the present study, 157(77.3%) patients were male. The median age was 88 (interquartile range; Q1-Q3 83–93). The median length of admission was 17(8, 33) days. Majority of the patients (*N* = 172, 84.7%) aged 80 years or older. CCI was 6.3(interquartile range; Q1-Q3 4.0, 8.0). The top 3 causes of death were malignant solid tumor (*N* = 74, 36.5%), infectious disease (*N* = 74, 36.5%), and cardiovascular disease (*N* = 23, 11.3%). Almost two thirds died of non-cancer diseases. Figure 1 presents the composition of the death cause.

### Clinical characteristics

Table 1 presents the comparison of clinical characteristics. There was no significant difference in age, gender, cause of death and functional status between the two groups (*p* > 0.05). The CCI was significantly lower in primary palliative care education group compared to those of usual care group (*p* < 0.05). In both groups, more than half were admitted more than once in their last year of life. About four-fifths of the surrogate was offspring who made CPR or DNR decisions.

**Table 1:** Comparison of clinical characteristics

	2014–2017 (*N* = 131)	2018–2019 (*N* = 72)	*χ*^*2*^/Z value	*p*-value
Age *N* (%)				
< 80 years	21 (16.0)	10 (13.9)	4.043	0.132
80 ~ 89 years	65 (49.6)	27 (37.5)		
≥ 90 years	45 (34.4)	35 (48.6)		
Gender *N* (%)				
Male	100 (76.3)	57 (79.2)	0.212	0.645
Female	31 (23.7)	15 (20.8)		
Cause of death *N* (%)				
Cancer	55 (42.0)	27 (37.5)	0.429	0.807
Infectious disease	47 (35.9)	27 (37.5)		
Others	29 (22.1)	18 (25.0)		
Times of hospitalization in past 12 months N (%)				
1	50 (38.2)	18 (25.0)	3.756	0.153
2 ~ 3	50 (38.2)	32 (44.4)		
≥ 4	31 (23.6)	22 (30.6)		
ADL score *N* (%)				
≥ 75	15 (11.5)	8 (11.1)	0.011	0.995
50 ~ 70	27 (20.6)	15 (20.8)		
≤ 45	89 (67.9)	49 (68.1)		
CCI	6.0 (5.0, 8.0)	5.0 (3, 7.8)	-3.427 (Z value)	0.001
Persons who sigh CPR *N* (%)				
Offspring	110 (84.0)	56 (77.8)	1.286	0.609
Spouse	16 (12.2)	11 (15.3)		
Others	3 (2.3)	2 (2.8)		
No CPR signature	2 (1.5)	3 (4.1)		
Preference of CPR *N* (%)	(missing cases = 2)	(missing cases = 3)		
DNR	107 (82.9)	57 (82.6)	1.533	0.465
Full or partial consensus	14 (10.9)	5 (7.2)		
CPR to DNR	8 (6.2)	7 (10.2)		

### Medical resource use and life-sustaining treatment

Table 2 presents the use of medical resources and LST. In primary palliative care education group, pain controlling drugs were used more (*p* < 0.05). The number of oral drugs decreased (4.9 ± 4.1 vs. 6.2 ± 4.6), but there was no statistical difference (*p* = 0.05). There was no difference in length of hospitalization, ICU admission, polypharmacy, use of broad-spectrum antibiotics, blood infusions, albumin infusions, nasogastric/nasoenteric tubes, parenteral nutrition, renal replacement, mechanical ventilation and puncture (*p* > 0.05). In the usual care group, percentage of invasive and non-invasive ventilation was 18.3% (*N* = 24) and 16.8% (*N* = 22), respectively. While, in the primary palliative care education group, that was 9.7% (*N* = 7) and 16.7% (*N* = 12). Nevertheless, the total ventilation percentage did not differ statistically.

**Table 2:** Comparison of medical resources and life-sustaining treatment

	2014–2017 (*N* = 131)	2018–2019 (*N* = 72)	*χ*^*2*^/Z value/ *t* value	*p*-value
Length of hospitalization (Days/IQR)	18.0 (8, 33)	15.5 (8, 34)	-0.517 (Z value)	0.605
ICU admission (Yes *N*/%)	23 (17.6)	11 (15.3)	0.173	0.677
Numbers of oral drugs (*Mean* ± *SD*)	6.2 ± 4.6	4.9 ± 4.1	1.967 (t value)	0.05
Polypharmacy (Yes *N*/%)	83 (62.6)	37 (51.4)	2.422	0.120
Pain controlling drugs (Yes *N*/%)	18 (13.7)	21 (29.2)	6.144	0.013
Broad-spectrum antibiotics (Yes *N*/%)	91 (69.5)	53 (73.6)	0.387	0.534
Blood infusion (Yes *N*/%)	52 (39.7)	35 (36.1)	0.252	0.616
Albumin infusion (Yes *N*/%)	97 (74.0)	46 (63.9)	2.302	0.129
Nasogastric/nasoenteric tube (Yes *N*/%)	98 (74.8)	49 (68.1)	1.061	0.303
Nutrition route (*N*/%)				
Oral feeding	27 (20.6)	13 (18.1)	0.217	0.897
Nasogastric/nasoenteric tube	41 (31.3)	24 (33.3)		
Parenteral nutrition	63 (48.1)	35 (48.6)		
Puncture (Yes *N*/%)	21 (16.0)	15 (20.8)	0.735	0.391
Renal replacement (Yes *N*/%)	8 (6.1)	4 (5.6)	0.025	0.873
Intubation (Yes *N*/%)	18 (13.7)	6 (8.3)	1.303	0.254
Mechanical ventilation (Yes *N*/%)	46 (35.1)	19 (26.4)	1.625	0.202
Trachoestomy (Yes *N*/%)	4 (3.1)	0 (0.0)	2.243	0.134

### Cardiopulmonary resuscitation attempts before death

Table 3 presents CPR attempts before death. Fewer patients of primary palliative care group received electric defibrillation, bag mask ventilation and vasopressor (*p* < 0.05). The ratio of chest compression and respiratory stimulants use decreased in PPC education group, yet there was no statistical difference compared with the usual care group (*p* > 0.05).

**Table 3:** Comparison of CPR attempts before death

	2014–2017 (*N* = 131)	2018–2019 (*N* = 72)	*χ*^*2*^	*p*-value
Chest compression (Yes *N*/%)	16 (12.2)	5 (6.9)	1.391	0.238
Electric defibrillation (Yes *N*/%)	6 (4.6)	0 (0.0)	5.356	0.021
Bag mask ventilation (Yes *N*/%)	33 (25.2)	9 (12.5)	4.560	0.033
Vasopressor (Yes *N*/%)	118 (90.1)	56 (77.8)	5.739	0.017
Respiratory stimulant (Yes *N*/%)	83 (63.4)	36 (50.0)	3.418	0.064

## Discussion

### Main findings

This study explored the effect of PPC education on LST intensity in EOL care for inpatients in the department of geriatrics. Most of the patients aged 80 years and older (84.7%). The leading causes of death were malignant solid tumors (36.5%) and infectious diseases (36.5%). After PPC education, pain controlling drugs were used more, while electric defibrillation, bag mask ventilation and vasopressors were used less. There was no significant change in the use of blood infusions, albumin infusions, kidney replacement treatments, mechanical ventilation, nasogastric/nasoenteric tubes and parenteral nutrition. This suggests that our PPC education partially changed LST intensity in EOL care with improvements in symptom management and avoidance of inappropriate CRP attempts.

### Symptom assessment and management skills are the key contents of PPC education

The key components of EOL care include symptom management and communication with patients and family members about advance care planning (ACP). This helps clinicians to understand patient’s and their family member’s thoughts on LST and code status. On one hand, it is necessary to effectively manage patient symptoms by providing appropriate pain relief and timely procedures to alleviate discomfort. On the other hand, a good quliaty of death involves the discontinuation of futile medications and avoiding high intensity treatments that prolongs or increases pain, such as ICU admission and unnecessary use of LST. In this study, after PPC education, more pain controlling drugs were used (from 13.7% to 29.2%). This suggests that our geriatricians and nurses were more aware of symptom management in terminally ill patients. Several reasons may be attributed to this improvement. Firstly, from department perspective, we recognized the need for PPC education for medical staff in the geriatrics department. Therefore, we participated in a national education program and hosted subsequent lectures and were required to self-study. At the same time, as a teaching hospital, two doctors in our department gave palliative care lessons to postgraduate students as teaching benefits teachers as well as students. Thus knowledge and skills of symptom management may have improved. Secondly, from a whole hospital management level, all staff were required to undertake training of cancer pain management and pain assessment in medical records were requested when prescribing pain controlling drugs. One study found negative association between opioid underuse and formal pain assessment [14]. All above reasons may have contributed to the improvement of pain control. A report from Netherland [15] showed a mean number of nine medications prescribed in the last week before death. In our study the number of oral medications dropped from six to five after PPC education. Medications added to treat pain and other physical symptoms would be reasonable for patients with a serious or advanced illness. However, preventive medications and other futile medications should be deprescribed [16].

Pain, discomfort, difficulty swallowing, loss of well-being, depression, delirium are the main problems for older adults at the EOL [17, 18]. The prevalence of pain and difficulty swallowing was 52–90 and 58–81% respectively [17]. In this study, the percentage of nasogastric/nasoenteric tube placement was 74.8% and 68.1% in the two groups, which was higher than another study [19]. It suggested high prevalence of dysphagia at the EOL in older adults. At present, there is a lack of hospice institutions in China resulting in patients with advanced cancer or end-stage chronic diseases still visiting the emergency department or requiring beds in a specialty department in tertiary hospitals. It is therefore necessary to carry out PPC education in these department including formal symptom assessments and EOL management skills.

### More effort should be put to reduce futile CPR at the end of life

In our study, the percentage of chest compression attempts was correlated to CPR willingness. After PPC education, we used less electric defibrillation, bag mask ventilation and vasopressors. This suggests an improvement in medical staff awareness of DNR and their abilities to identify inappropriate CPR. A study [11] found the last CPR attempt among patients 80 years or older was perceived as appropriate by 52.4% of the clinicians, 29.1% were uncertain about the appropriateness and 18.5% perceived the CPR attempt as inappropriate. 89.2% CPR attempts involved non-shockable rhythms. The likelihood of cardiac arrest in older adults with non-shockable rhythms increases with age. This may explain decreased use of defibrillation in advanced age. However, some patients selected DNR, bag mask ventilation, vasopressor and respiratory stimulants were still used. When a patient has a cardiac arrest or stops breathing, the clinician may start resuscitation and administrate vasopressors as routine treatment. However, CPR is not appropriate for patients with a terminal illness or those who are dying. If a patient is considered to be in the dying process (assessed by prognostic tools in combination with the clinician’s judgement), futile treatments should be avoided. Prognostication involves 3 key components: formulation of the patient’s prognosis; communication of the patient’s prognosis; and the patient or surrogate’s interpretation of the communicated prognosis [20]. A study [19] had shown that early DNR consent can reduce the CPR attempt and other invasive treatments in patients with advanced cancer. The patients are more willing to accept a DNR decision when it was referred to as ‘allowing natural death’, when there is comprehensive information and when there are worse outcomes [21]. Our findings have implications for further PPC education in geriatrics which focus on CPR appropriateness, skills of prognosis and code status discussion.

Our study found code status decision was made mainly by patient’s children. This may be related to Chinese traditional culture of close relationships among family members. On the other hand, the median age of participants was 88 years old, some of the older adults lose capacity to make medical decisions at the EOL. According to Chinese tradition and routine communication pattern, medical staff first ask the patient’s surrogate if the patient had living will. When the answer is ‘no’, then ask the surrogate whether patient or he/she make the decision about CPR. In most cases, the surrogate is patient’s children, CPR or DNR was determined by them ultimately. In our study, the percentage of full consent and partial consent of CPR before and after PPC education was 10.9 and 7.2% respectively. Another survey of ACP attitudes from mainland China [22] found that only 43.6% non-cancer older adults choose DNR, which was lower than that of our study. This data gap can be explained by different participants resulting in different disease severities and age. We should also note that in our study, 6.2 and 10.2% of patients in the two groups changed their CPR preferences from consent to disapproval. This shows that patients can change their treatment preferences. As the disease advances, medical staff should have full and timely communication with the patients and their families. Terminally ill patients or their relatives tend to accept DNR. Age is an important determinant for the initiation of DNR orders in critically ill patients, older adults aged 75 to 84 and ≥ 85 years have greater DNR orders compared with those < 65 years [23]. Therefore, medical staff should identify the patients who are at the EOL and provide adequate information to the patient and their relatives, explaining their prognosis in order to avoid futile treatments.

### Primary palliative care education about end-stage non-cancer disease may reduce life-sustaining treatment in geriatrics

In this study, the application of some LST had not been significantly reduced after PPC education. It may be due to three reasons. Firstly, it may be related to the causes of death in our study. Of all the participants, approximately one third died of malignant tumors and two thirds died of non-cancer diseases. Disease diagnosis (ischemic heart disease, infectious disease, chronic lung disease, chronic heart disease) can impact the intensity of EOL care among older adults [8]. Secondly, as a tertiary hospital in a capital city, antibiotics and blood products are relatively easy to obtain and life support techniques are widely used. A study [8] from Korean found large hospitals and facilities located in metropolitan areas were significantly associated with increased likelihood of receiving high-intensity EOL care. Thirdly, there is a lack of palliative care criteria for non-cancer older adults in mainland China.

Older adults usually have complex conditions involving comorbidity, disability, frailty, malnutrition and impaired cognition. LST preferences should be discussed as early as possible during EOL care [24]. Late DNR consent is associated with patients receiving unnecessary medical interventions to sustain life. Providing patients and their families with comprehensive information on LST can help to reduce EOL care intensity. A retrospective cross-sectional study [21] from the United States compared the quality of death between patients with end-stage renal disease, cardiopulmonary failure and frailty vs patients with cancer and dementia. The study showed that the former group had reduced quality of death with less palliative care, less DNR and more ICU admission. We should thus focus on patients with end-stage non-cancer diseases and more palliative care education should be carried out with related issues explored.

## Conclusion

In conclusion, our study found that PPC education promoted pain controlling drug use and DNR implementation, but does not reduce all LST in our department. It suggests we should put more efforts on education about symptom assessment and management skills, prognostication, ACP and code status discussion and end stage non-cancer disease management in the future. There are limitations to our study. Firstly, it is a retrospective study, information is limited to the contents of case records. We did not include multidimensional aspect of pain control, the spiritual and psychological aspects of pain control, holistic assessment and other symptoms management. In future work, we will use more assessment scales such as Edmonton Symptom Assessment Scale, Integrated Palliative Care Outcome Scale, Numerical Rating Scale to evaluate symptom burden and effects of interventions. Secondly, it is a single-center research. Our hospital is a tertiary hospital without hospice wards. It does not represent the status of primary health care institutions or hospices. However, it can be used as an example experience for other tertiary hospitals in China. Thirdly, knowledge about ACP and DNR of patients and their families may have improved naturally over time. This effect was not corrected for in our study. At present, there is a huge gap between the supply and demand of palliative care in China. PPC education and research should be carried out in geriatrics and other departments, especially for non-cancer diseases at the end of life. In future, PPC education should focus on management of pain and other symptoms in details, basic discussion skills about prognosis and communication skills of goals of treatment. It would also be interesting to improve PPC education content and pattern. We should further develop research to evaluate the effect of education programmes on clinical practice and if PPC education improve quality of end-of-life.

## Data Availability

The data collection transcripts are available on reasonable demand to the corresponding author.

## Abbreviations

EOL: End-of-life
SPC: Specialty Palliative Care
PPC: Primary Palliative Care
LST: Life-sustaining Treatment
CPR: Cardiopulmonary Resuscitation
DNR: Do-not-resuscitate
ADL: Activity of Daily Living
ICU: Intensive Care Unit
CCI: Charlson Comorbidity Index
ACP: Advance Care Planning
